# Coconut shell derived biochar to enhance water spinach (*Ipomoea aquatica* Forsk) growth and decrease nitrogen loss under tropical conditions

**DOI:** 10.1038/s41598-019-56663-w

**Published:** 2019-12-30

**Authors:** Fengliang Zhao, Ganghua Zou, Ying Shan, Zheli Ding, Minjie Dai, Zhenli He

**Affiliations:** 1 Institute of Environmental and Plant Protection, Chinese Academy of Tropical Agricultural Sciences (CATAS), Haikou, 571101 Hainan China; 2 National Agricultural Experimental Station for Agricultural Environment, Danzhou, 571737 Hainan P.R. China; 3 Haikou Experimental Station, Chinese Academy of Tropical Agricultural Sciences (CATAS), Haikou, 571101 Hainan China; 4 Indian River Research and Education Center, Institute of Food and Agricultural Sciences, University of Florida, Fort Pierce, FL USA

**Keywords:** Environmental chemistry, Environmental impact

## Abstract

Farms usually apply excessive nitrogen (N) fertilizers, especially in a vegetable production system, resulting in severe N leaching loss. Although there have been some reports on the impact of biochar on the N leaching in farmlands, most of them focused on field crops in temperate or subtropical religions. Limited information about N leaching in the tropical vegetable system is available regarding the quantitative data and effective countermeasures. A field experiment was conducted to quantify N leaching in a tropical leafy production system (*Ipomoea aquatica* Forsk) and to evaluate the effects of coconut shell biochar on N loss and crop growth. The results showed that compared to conventional fertilization with the 240 kg N ha^−1^ application rate (NPK), biomass yield of water spinach increased by 40.1% under the high biochar application rate of 48 t ha^−1^ (HBC), which was significantly higher than that of NPK treatment. Moreover, The HBC treatment decreased N leaching by 34.0%, which can be attributed to enhanced crop uptake which increased by 40.3% as compared to NPK treatment. The NH_4_^+^/NO_3_^−^ ratio in leachates was between 0.01 and 0.05. It was concluded that coconut shell derived biochar improved the biomass yields of water spinach and reduced the leaching N loss, which provides a promising amendment in tropical regions.

## Introduction

Excessive chemical fertilizers are often applied to maintain or enhance agricultural productivity under the pressures of shrinking land area per capita. The average chemical fertilizer usage per hectare in China has increased from 86.7 kg ha^−1^ in 1980 to 359 kg ha^−1^ in 2016, about 3.3 times that of the United States and 3.6 times that of the world average^1^. The average N input for vegetable crops was raised to 588 kg ha^−1^ according to the 2013–2015 survey, which was 2.7 times the recommended for open-field vegetables in China, respectively^2,3^. Excessive chemical fertilizers have caused many environmental problems, including soil acidification, water and air pollution^4,5^. One of the most common environmental concerns is NO_3_^−^ leaching into underground water and caused great risk for human health^6,7^.

Biochar is a carbon-rich product made by pyrolysis of organic matter under partial or complete exclusion of oxygen^8^. Biochar can be produced from various feedstocks. It is composed of condensed aromatic groups that are partially responsible for its highly biochemical recalcitrance than many other forms of organic matter in soil^9^. Biochars are highly porous, usually alkaline, and exhibit large specific surface area^10^. Owing to these inherent physicochemical properties, biochar affects many soil properties including soil pH, organic matter, water holding capacity and microbial composition and diversity^11,12^. In addition, oxidation of biochar at a slow rate in soil leads to the production of negatively-charged functional groups, such as carboxyl and phenolic groups^13,14^. The presence of these groups implies a high density of functional groups at the surface of biochar particles that interact with nutrient ions by electrostatic, complexation, or capillary forces^15,16^. These properties of biochars can reduce the leaching of nutrients from soil and subsequent accessibility of nutrients to crops^17^.

As a promising soil amendment, there has been an increased interest in biochar research in recent years due to its potential beneficial effects on soil properties and water remediation^18,19^. It has the potential to be a low cost and efficient sorbent for NH_4_^+^ and NO_3_^−^^20^ and may reduce N leaching and increase crop yields^21,22^. NH_4_^+^ could be adsorbed on the surface of biochar by cation exchange and entrapped in its pore structures. Furthermore, NO_3_^−^ could be also adsorbed as a result of exchange reactions between base functional groups^20,23,24^. However, biochar addition to soil showed inconsistent results on crop growth, soil properties and nutrient N leaching^10,23–26^ because biochar properties vary widely, depending on the biomass source, pyrolysis temperature (350–900 °C) and application rate (4–90 t ha^−1^)^8,20,27,28^.

In many tropical regions, organic wastes from the processing of coconut are widely generated and mostly they are destined for landfills^29^. An alternative way to reuse the coconut shells may be the production of biochar to keep a sustainable organic matter cycle. Water spinach (*Ipomoea aquatica* Forsk) is a kind of common leafy vegetables in the South of China. There is limited information about the impacts of biochar on water spinach and tropical vegetable production system. The present research was aimed at (1) comparing the efficiency of soil amendments on the growth of water spinach; (2) determining whether biochar could effectively reduce N leaching.

## Results

### Growth of water spinach and N use efficiency

**Figure 1:**
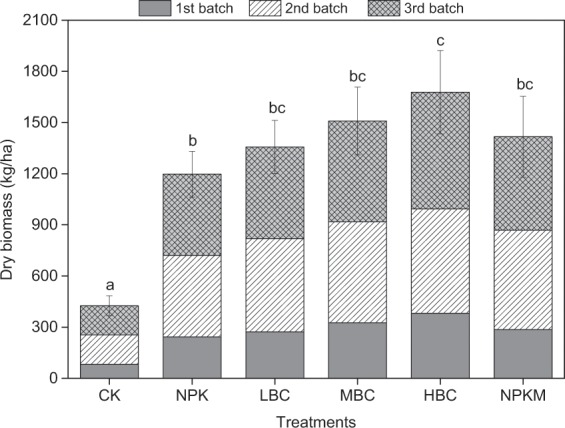
Harvested biomass yields of water spinach during the experiment. The same letter on the top of vertical bars indicates no significant difference in the accumulative dry biomass yield of water spinach at the 0.05 level. CK-control (without N fertilizer and soil amendments); NPK- conventional chemical fertilization; LBC, MBC and HBC- chemical fertilizer (NPK) plus 12 t ha^−1^, 24 t ha^−1^, 48 t ha^−1^ biochar, respectively; NPKM-NPK plus 12 t ha^−1^ manure.

The biomass of water spinach was harvested three times during the experiment (Fig. 1). The first harvest had obvious biomass due to the short growth period (26 days) than that of the other two times (33 days). Significant differences occurred in every harvest dry biomass between the treatments. Biochar amendment promoted the growth of water spinach. The accumulative dry biomass yields of spinach amended with biochar was from 1357 to 1678 kg ha^−1^, which was 13.3–40.1% more than that of chemical fertilizer treatment (NPK, 1197 kg ha^−1^). The difference in biomass yield between the treatments of NPK plus a high biochar rate (HBC, 48 t ha^−1^) and NPK was significant (P ≤ 0.05), whereas the difference was not significant when manure was applied, although the increase was 18%, as compared to NPK treatment.

**Figure 2:**
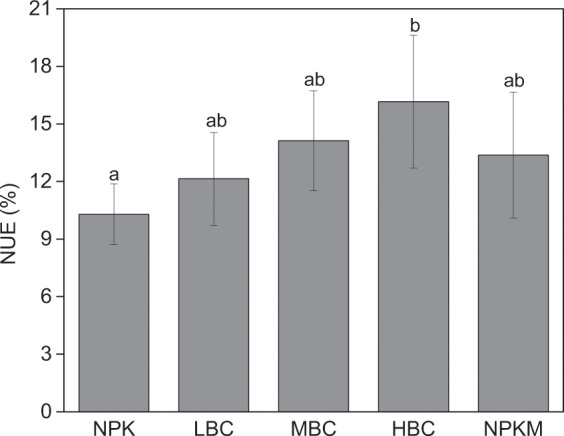
N use efficiency (NUE) by water spinach with different treatments. Different lowercase letters on the top of vertical bars indicate significant differences between treatments (*P* < 0.05). CK-control (without N fertilizer and soil amendments); NPK- conventional chemical fertilization; LBC, MBC and HBC- chemical fertilizer (NPK) plus 12 t ha^−1^, 24 t ha^−1^, 48 t ha^−1^ biochar, respectively; NPKM-NPK plus 12 t ha^−1^ manure.

Biochar addition increased N use efficiency (NUE) by water spinach (Fig. 2). The NUE was ~6% higher with biochar at the high application rate than that of NPK treatment (P ≤ 0.05), and there were significant differences between them. Medium rate of biochar had a similar NUE of NPKM, and slightly higher than that of low biochar rate.

### N leaching

**Figure 3:**
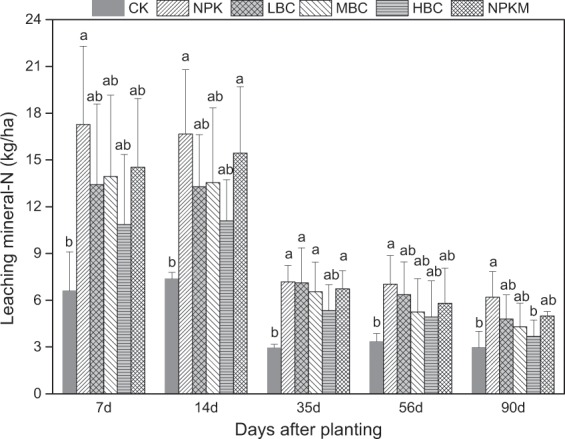
The leaching mineral-N as affected by different treatments during the water spinach growing season. Different lowercase letters on the top of vertical bars indicate significant differences between treatments (*P* < 0.05). CK-control (without N fertilizer and soil amendments); NPK- conventional chemical fertilization; LBC, MBC and HBC- chemical fertilizer (NPK) plus 12 t ha^−1^, 24 t ha^−1^, 48 t ha^−1^ biochar, respectively; NPKM-NPK plus 12 t ha^−1^ manure.

**Figure 4:**
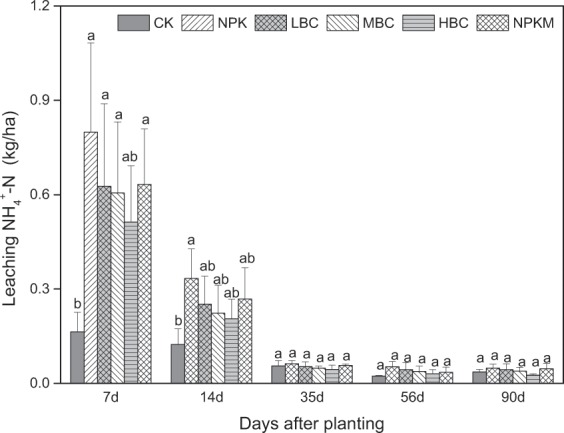
The leaching NH_4_^+^-N as affected by different treatments during the water spinach growing season. Different lowercase letters on the top of vertical bars indicate significant differences between treatments (*P* < 0.05). CK-control (without N fertilizer and soil amendments); NPK- conventional chemical fertilization; LBC, MBC and HBC- chemical fertilizer (NPK) plus 12 t ha^−1^, 24 t ha^−1^, 48 t ha^−1^ biochar, respectively; NPKM-NPK plus 12 t ha^−1^ manure.

**Figure 5:**
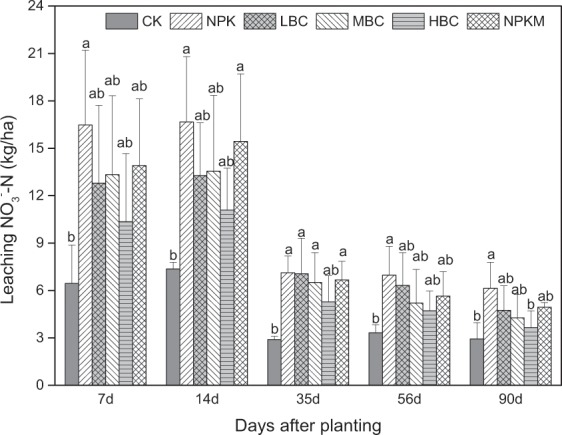
The leaching NO_3_^−^-N as affected by different treatments during the water spinach growing season. Different lowercase letters on the top of vertical bars indicate significant differences between treatments (*P* < 0.05). CK-control (without N fertilizer and soil amendments); NPK- conventional chemical fertilization; LBC, MBC and HBC- chemical fertilizer (NPK) plus 12 t ha^−1^, 24 t ha^−1^, 48 t ha^−1^ biochar, respectively; NPKM-NPK plus 12 t ha^−1^ manure.

The amount of leached N from different treatment was determined by its concentration in the leachate multiplied with the amount of leachate. The cumulative amount of leached N reflected the concentration of N in the leachate and the leachate volume. Due to minor differences in leachate volume among all the treatments (Supplementary Fig. 1), the leaching N was more related to mineral N concentration (Supplementary Fig. 2). The amount of leached N was highest in the 7 days after planting before root systems of water spinach established (Fig. 3). Afterwards, leachate NO_3_^−^-N and NH_4_^+^-N declined with the growth of water spinach (Figs. 4 and 5). The accumulative leachate N during the experiment was the highest for NPK (60.3 kg ha^−1^), 34.0% higher than that of HBC (39.5 kg ha^−1^).

Regardless of treatments and days after planting, more N in the leachate occurred as NO_3_^−^ than NH_4_^+^ (Supplementary Figs. 3 and 4). The NH_4_^+^/NO_3_^−^ ratio in the leachate was from 0.01 to 0.05. For NPK, the NO_3_^−^-N concentration in the leachate was 42.5 mg L^−1^ in the initial leachate and 16.4 mg L^−1^ at the end of the experiment. These values were 30.3% and 39.0% higher than that of HBC during the corresponding periods.

### Soil mineral N

**Figure 6:**
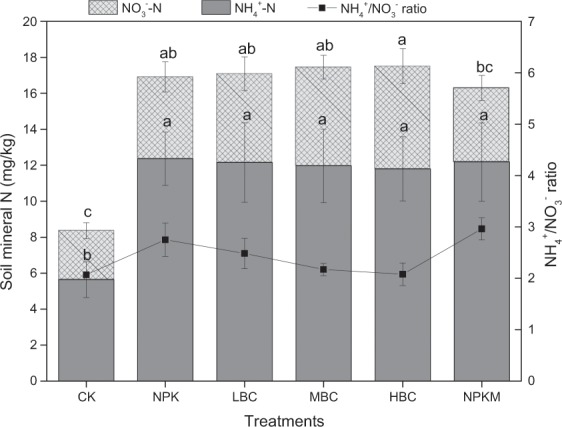
Soil mineral N and NH_4_^+^/NO_3_^−^ ratio at the end of the experiment. Different lowercase letters on the top of vertical bars indicate significant differences between treatments (*P* < 0.05). CK-control (without N fertilizer and soil amendments); NPK- conventional chemical fertilization; LBC, MBC and HBC- chemical fertilizer (NPK) plus 12 t ha^−1^, 24 t ha^−1^, 48 t ha^−1^ biochar, respectively; NPKM-NPK plus 12 t ha^−1^ manure.

Soil mineral N content at the 0–30 cm soil depth before the experiment was 8.82 mg kg^−1^ and decreased to 6.81 mg kg^−1^ for CK at the end of the experiment. Soil NO_3_^−^-N content for HBC treatment was 5.72 mg kg^−1^ and was significantly higher than CK and NPKM treatments (Fig. 6). Soil NH_4_^+^-N content was from 5.64 mg kg^−1^ to 12.4 mg kg^−1^; there was no significant difference between the fertilization treatments. The NH_4_^+^/NO_3_^−^ ratios of biochar treatments (2.1–2.5) were lower than those of NPK and NPKM treatments (2.8–3.0).

### N balance

The N balance in the open-field water spinach production system was greatly affected by the treatments (Table 1). N input (which was the sum of N from fertilizer, soil mineralization, and mineral N before planting) ranged from 64.3 kg ha^−1^ for CK to 304 kg ha^−1^ for the other treatments. Fertilizer was the dominant N input. Compared with NPK, N output of biochar treatments increased by 5.27%-18.4%.

**Table 1:** The amounts of N losses (kg ha^−1^) in the tropical water spinach production system as affected by biochar amendment.

Items		Treatments					
		CK	NPK	LBC	MBC	HBC	NPKM
Input	Urea	0.00 ± 0.00 b	240 ± 0.00 a	240 ± 0.00 a	240 ± 0.00 a	240 ± 0.00 a	240 ± 0.00 a
	Mineralization	25.9 ± 3.91 a	25.9 ± 3.91 a	25.9 ± 3.91 a	25.9 ± 3.91 a	25.9 ± 3.91 a	25.9 ± 3.91 a
	0–30 cm soil mineral N at the beginning of experiment	38.4 ± 6.23 a	38.4 ± 6.23 a	38.4 ± 6.23 a	38.4 ± 6.23 a	38.4 ± 6.23 a	38.4 ± 6.23 a
Output	Crop uptake	10.7 ± 1.81 c	35.1 ± 5.17 b	39.5 ± 6.95 ab	44.3 ± 7.25 ab	49.2 ± 9.55 a	42.5 ± 8.54 ab
	0–30 cm soil mineral N at the end of experiment	30.1 ± 5.16 b	60.9 ± 8.11 a	61.5 ± 10.9 a	62.9 ± 9.65 a	63.1 ± 9.48 a	58.7 ± 10.2 a
N loss	Apparent N loss	23.6 ± 2.49 b	208 ± 8.14 a	203 ± 11.4 a	197 ± 10.8 a	192 ± 13.4 a	203 ± 11.0 a
	N leaching loss	23.4 ± 3.38 b	54.7 ± 11.9 a	45.2 ± 13.6 ab	43.8 ± 15.5 ab	36.1 ± 12.2 ab	47.8 ± 11.4 a
	N leaching/Apparent N loss	99.6 ± 12.9 a	26.4 ± 6.59 b	22.5 ± 7.83 b	22.6 ± 9.34 b	19.2 ± 7.89 b	23.8 ± 7.06 b

Different lowercase letters on the top of vertical bars indicate significant differences between treatments (P < 0.05). CK-control (without N fertilizer and soil amendments); NPK- conventional chemical fertilization; LBC, MBC and HBC- chemical fertilizer (NPK) plus 12 t ha^−1^, 24 t ha^−1^, 48 t ha^−1^ biochar, respectively; NPKM-NPK plus 12 t ha^−1^ manure.

N losses caused by leaching ranged from 19.2 to 26.4%. In both years, N leaching loss was highest in the system managed according to the conventional farming practice (NPK) and was lowest in the system of HBC. Crop uptake N was highest for MBC, although there were no significant differences between fertilization treatments. Compared with NPK, biochar addition increased crop N uptake by 12.6–40.1%, reduced N leaching by 17.3–34.0%, and total N loss by 2.4–7.8%.

## Discussion

### Biochar increased vegetable yield in tropical religion

Biochar has been proven a renewable resource and eco-friendly material for improving soil fertility and increasing crop yields^30^. Although there are controversies regarding the extent and cause of positive benefits, biochar is widely used to boost crop yield^31^. In the tropics, water spinach commonly grows in acidic *Oxisols* with a low content of soil organic C and nutrients. Biochar amendment increased water spinach biomass by 13–40%, as compared to control (Fig. 1). The yield increase by biochar can be attributed to (i) increased nutrient supply for crop growth; (ii) enhanced water availability; (iii) improved soil quality; (iv) suppressing of soil-borne plant diseases^30,32,33^. Moreover, yield response to biochar has shown to be more evident on acid soils in the tropical regions than on neutral/alkaline soils in the temperate^34^. The global-scale meta-analysis by Jeffery *et al*. (2017) 31 indicated that biochar had on average no effect on crop yield in the temperate regions, yet elicited a 25% average increase in crop yield in the tropics. Moreover, the physicochemical properties of biochar also affected the extent of crop yield benefit^35^. For instance, the coconut shell biochar with higher pH and micropore specific surface area was more useful for improving soil fertility, nutrient availability and crop growth.

### N leaching in vegetable systems

Application rates of fertilizers in vegetable production systems often exceed crop requirements, resulting in a high accumulation of nutrients in the soil^36^. Soil NO_3_^−^ leaching was influenced by crop type, soil properties, irrigation management, and rainfall^37^. In this study, N leaching in the water spinach production system amounted to 36.1–54.7 kg ha^−1^ during the 90 days of the experiment (Fig. 5), which is consistent with the report by Chotangui *et al*.^38^. Wang *et al*.^39^ quantified the nitrate leaching by conducting a meta-analysis and pointed that NO_3_^−^-N leaching over the entire crop growing season in Chinese vegetable systems averaged 79.1 kg ha^−1^ under the high N inputs (on average 423 kg ha^−1^). Moreover, due to the low capacity to retain anions in soils, NO_3_^−^ is more prone to leaching than NH_4_^+^^40^. Therefore, NH_4_^+^ concentration in leachates was far lower than NO_3_^−^, and the NH_4_^+^/NO_3_^−^ ratio was only 0.01 to 0.05 (Figs. 5 and 6), which is consistent with the report by Zhang *et al*.^41^.

### Biochar decreased N leaching loss

Biochar addition has been shown to increase nutrient retention in highly weathered soils through different mechanisms including sorption of NO_3_^−^, NH_4_^+^ and organic-N; alteration of cation and anion exchange capacity and changes in microbial processes and activities^42^. Significant NO_3_^−^ adsorption occurred at pyrolysis temperatures ≥700 °C^20^. In the present study, as compared to control (application of 240 kg N ha^−1^ without biochar), biochar-amended treatment decreased N leaching by 17.3–34.0% (Fig. 5). Related research showed that negatively charged functional groups on biochar surface could adsorb positively charged NH_4_^+^^8,42^. It was reported that NO_3_^−^ retention driven by anion exchange with oxonium and pyridinium groups also occurred22. Kameyama *et al*.^43^ reasoned that the adsorption of NO_3_^−^ was a result of base functional groups and not a result of physical adsorption since surface area and micropore volumes followed different trends. Other mechanisms for reducing N leaching loss in the biochar-amended soils included enhanced N assimilation by crops and improved soil structure^44^. Enhanced N assimilation by water spinach was observed in the present study (Table 1). Biochar increased N uptake by 12.6–40.3%, as compared to control.

### NUE and N balance

The high-yield vegetable production systems in China were dependent on the intensive input of fertilizers over the past decades, especially N fertilizers, which have lowered NUE in croplands. In this present study, NUE of water spinach was 10.3–16.2% (Fig. 2), which is consistent with previous studies^45,46^, but lower than the average NUE of open-air and greenhouse vegetable production systems in China^47^.

Nitrogen balance, defined as the difference between N inputs and outputs, can be used as an indicator to reveal N loss in the vegetable cropping systems^47–49^. In this present study, N leaching accounted for 19.2–26.4% of total N losses (Table 1), and other loss could include runoff, NH_3_ volatilization, and gaseous emissions from nitrification and denitrification^50^. Previous studies indicated that NH_3_ volatilization loss to N input accounted for 18–24% of the total N input in the vegetable production systems^51^, and the proportion increased with the application of urea^23,52^. The nitrous oxide emissions (N_2_O) for vegetable fields were only around 0.94% of applied N fertilizer^53^.

## Conclusion

Concerns about nitrate contamination to groundwater and agricultural sustainable development have been impelling us to apply effective technologies to decrease N environmental loss and promote N use efficiency. In the present study, compared to conventional chemical fertilization treatment, biomass yield of water spinach increased by 13.3–40.1% with biochar amendment. Biochar addition also decreased N leaching by 17.3–34.0%, which is mainly attributed to enhanced crop uptake of N by 12.6–40.3% as compared to control. It was concluded that coconut shell derived biochar improved the biomass yields of water spinach and reduced the leaching N loss, which provides a promising amendment in tropical regions.

## Materials and Methods

### Experiment site

The field experiment was conducted on the Danzhou Experiment Station of Chinese Academy of Tropical Agricultural Science (19°30′36′′ N, 109°29′40′′ E). This region is characterized by a tropical monsoon climate with an average annual rainfall of 1815 mm and a mean annual temperature of 23.5 °C. The soil was derived from granite material, classified as *Oxisol* with a sandy loam texture. The surface soil (0–30 cm) had pH (H_2_O) 6.02, organic C 5.61 g/kg, total N, P and K of 0.96, 0.24 and 0.29 g kg^−1^, available N, P and K of 60.7, 9.28 and 41.6 mg kg^−1^.

### Experiment design and agricultural management practices

Water spinach (*Ipomoea aquatica* Forsk) is a kind of typical leafy vegetable in tropical region of China, which is most commonly grown in East, South and Southeast Asia. The experiment was conducted from July 1 to September 30, 2017, with three water spinach harvests. The seeds of water spinach (var. Ching Quat) were tested. The experiment was a completely randomized design with six treatments and three replications. Six treatments were: no N fertilizer and no soil amendments (CK), conventional chemical fertilizer (NPK), NPK plus a low biochar rate (LBC, 12 t ha^−1^), NPK plus a moderate biochar rate (MBC, 24 t ha^−1^), NPK plus a high biochar rate (HBC, 48 t ha^−1^) and NPK plus manure (NPKM, 12 t ha^−1^). Agricultural practices except for fertilizer management were identical for all the treatments. According to the local recommendation of leafy vegetable, all the treatments except for CK had the same N, P_2_O_5_ and K_2_O input, which were 240, 96 and 192 kg ha^−1^, respectively; 40% N, 100% P and 40% K are applied as base fertilizers by mixing with the topsoil (0–30 cm) and 100% amendments (biochar or manure) as urea, calcium superphosphate and potassium sulfate. 30% N and 30% K are applied after the second and third harvest, respectively. Coconut shells were crushed and then were pyrolyzed in a sealed stove. The pyrolysis temperature was raised to 700 °C at a ramp rate of 20 °C min^−1^ and lasted for 2 h. The biochar had a pH (H_2_O) 10.2, content of C 65.7%, H 2.43%, N 0.55%, S 0.08%, P 0.16%, K 1.11% and micropore specific surface area 152.8 m^2^/g. Manure with 21% C, 2.2% N, 1.2% P and 1.4% K was acquired from a local fertilizer shop. The experiment consisted of 18 field plots (each 2.0 × 2.0 m), which were separated from each other by an anti-seepage film and cement bricks (600-mm depth) to prevent lateral water movement.

### Collection of soil and plant samples and measurements

All the aboveground tissues of water spinach were harvested three times during the experiment and the harvested biomass from each plot was weighed. The subsamples were oven-dried to a constant weight at 75 °C to calculate the water content of biomass, and the dried plant samples were powdered and analyzed for total N concentration using a CHNS element analyzer (Vario EL III, Elementary, Hanau, Germany)^54^.

After harvest, the 0–30 cm soil layer was sampled using a stainless steel auger (15 mm interior diameter). Soil pH was determined in water at the 1:2.5 soil to solution ratio. Mineral N in soil (NH_4_^+^-N and NO_3_^−^-N) was extracted by 1 M KCl and NH_4_^+^-N concentration in extracts was determined with the colorimetric-indophenol blue method^55^ and NO_3_^−^-N concentration using a dual-wavelength spectrophotometry (Hitachi U-2100; Hitachi, Tokyo, Japan)^56^.

### Soil leachate sampling and analysis

Eighteen lysimeters (one per plot) were installed in the field before the basal fertilizer was applied^57^. Each lysimeter consisted of a 350-mm-diameter polyvinyl chloride (PVC) cover and a plastic bucket. The PVC cover was at 600-mm-depth below the ground with holes, filtering mesh and sandy gravels filled up to 50-mm-depth. The plastic bucket was used for collecting leachates. This allowed soil solution to drain from the sandy gravel cover and to be collected as leachate in the buckets by vacuum pump. The leachate was collected on day 7, 14, 35, 56 and 90. Total leachate volume was recorded, and subsamples were analyzed for NH_4_^+^-N and NO_3_^−^-N concentration with the colorimetric-indophenol blue method and dual-wavelength spectrophotometry, respectively.

### Data analysis

N use efficiency (NUE) of water spinach was calculated with the following equation^58^:

$${\rm{NUE}}=({{\rm{N}}}_{{\rm{treat}}}-{{\rm{N}}}_{{\rm{control}}})/{{\rm{N}}}_{{\rm{fert}}}.$$

where N_treat_ and N_control_ are crop N uptake by the aboveground biomass with and without N fertilization, respectively. N_fert_ is N application rate.

Apparent N loss from the water spinach production system was calculated according to Widowati *et al*. and Zhang *et al*.^59^:

$${\rm{Apparent}}\,{\rm{N}}\,{\rm{loss}}={{\rm{N}}}_{{\rm{initial}}}+{{\rm{N}}}_{{\rm{fert}}}+{{\rm{N}}}_{{\rm{\min }}}-{{\rm{N}}}_{{\rm{end}}}\,\mbox{--}\,{{\rm{N}}}_{{\rm{crop}}}$$

where N_initial_ and N_end_ are soil mineral N (sum of NH_4_^+^ and NO_3_^−^) at the 0–30 cm depth at the initial and end of the experiment. N_crop_ is the N uptake by the aboveground of water spinach. N_min_ is the soil N mineralized within the vegetable growing period, and was calculated as follows^41^:

$${{\rm{N}}}_{{\rm{\min }}}=({{\rm{N}}}_{{\rm{end}}}+{{\rm{N}}}_{{\rm{crop}}}+{{\rm{N}}}_{{\rm{leaching}}}-{{\rm{N}}}_{{\rm{initial}}}-{{\rm{N}}}_{{\rm{fert}}})\,{\rm{of}}\,{\rm{no}}\,{\rm{N}}\,{\rm{application}}\,{\rm{treatment}}.$$

All data were analyzed by one-way analyses of variance (ANOVAs) with SPSS (version 16.0). When an ANOVA was significant, means were compared using the Duncan multi-range test. The significant level was set at *P* < 0.05. Figures and tables were generated with Microsoft Excel 2010 and Origin 8.0. The data were expressed as mean ± standard deviation (*n* = 3).

## Supplementary information

Supplementary Information File 1
