# Supplementary material for: Coconut shell derived biochar to enhance water spinach (Ipomoea aquatica Forsk) growth and decrease nitrogen loss under tropical conditions
Source: Sci Rep. 2019 Dec 30;9:20291. doi: 10.1038/s41598-019-56663-w (PMC6937338; doi:10.1038/s41598-019-56663-w)
Supplement: Supplementary file 1 — Supplementary Information File 1 [file 41598_2019_56663_MOESM1_ESM.docx]

**Coconut shell derived biochar to enhance water spinach (*Ipomoea aquatica* Forsk) growth and decrease nitrogen loss under tropical conditions**

Fengliang Zhao^1,2*^, Ganghua Zou^1,2^, Ying Shan^1,2^, Zheli Ding^3^, Minjie Dai^1^ & Zhenli He^4^

^1^ Institute of Environmental and Plant Protection, Chinese Academy of Tropical Agricultural Sciences (CATAS), Haikou 571101, Hainan, China.

^2^ National Agricultural Experimental Station for Agricultural Environment, Danzhou 571737, Hainan, P.R. China.

^3^ Haikou Experimental Station, Chinese Academy of Tropical Agricultural Sciences (CATAS), Haikou, 571101, Hainan, China.

^4^ Indian River Research and Education Center, Institute of Food and Agricultural Sciences, University of Florida, Fort Pierce, FL, USA.

^*^Fengliang Zhao and Ganghua Zou contributed equally to this work. Correspondence and requests for materials should be addressed to F.Z. (email: fengliangzhao@catas.cn)

**Supplementary Material**

**Supplementary Fig. 1** Leachate volume of different treatments in lysimeters during the water spinach growing season.


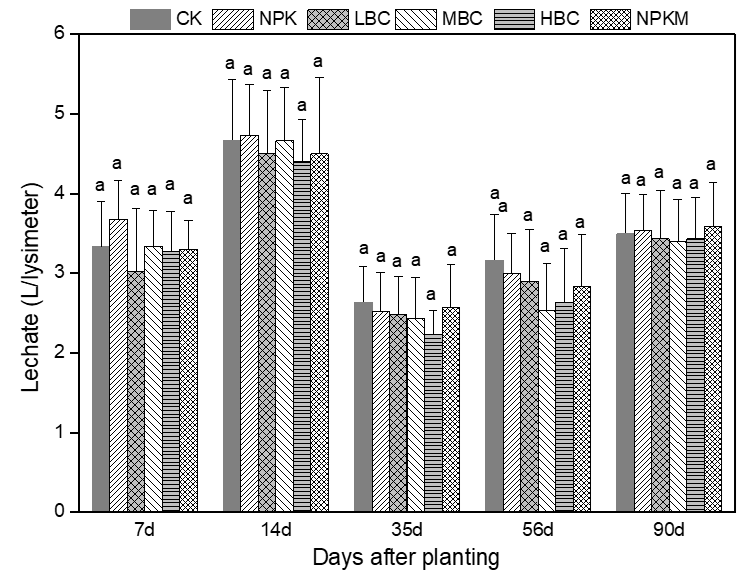


CK-control (without N fertilizer and soil amendments); NPK- conventional chemical fertilization; LBC, MBC and HBC- chemical fertilizer (NPK) plus 12 t ha^-1^, 24 t ha^-1^, 48 t ha^-1^ biochar, respectively; NPKM-NPK plus 12 t ha^-1^ manure. The same lowercase letter on the top of vertical bars after different planting days indicates significant differences between treatments (*P* ≥ 0.05).

**Supplementary Fig. 2** Mineral N concentration in the leachate during the water spinach growing season.

CK-control (without N fertilizer and soil amendments); NPK- conventional chemical fertilization; LBC, MBC and HBC- chemical fertilizer (NPK) plus 12 t ha^-1^, 24 t ha^-1^, 48 t ha^-1^ biochar, respectively; NPKM-NPK plus 12 t ha^-1^ manure. Different lowercase letters on the top of vertical bars indicate significant differences between treatments (*P* <0.05).

**Supplementary Fig. 3** Nitrate N (NO_3_^-^-N) concentration in the leachate during the water spinach growing season.

CK-control (without N fertilizer and soil amendments); NPK- conventional chemical fertilization; LBC, MBC and HBC- chemical fertilizer (NPK) plus 12 t ha^-1^, 24 t ha^-1^, 48 t ha^-1^ biochar, respectively; NPKM-NPK plus 12 t ha^-1^ manure. Different lowercase letters on the top of vertical bars indicate significant differences between treatments (*P* <0.05).

**Supplementary Fig. 4** Ammonium N (NH_4_^+^-N) concentration in the leachate during the water spinach growing season.

CK-control (without N fertilizer and soil amendments); NPK- conventional chemical fertilization; LBC, MBC and HBC- chemical fertilizer (NPK) plus 12 t ha^-1^, 24 t ha^-1^, 48 t ha^-1^ biochar, respectively; NPKM-NPK plus 12 t ha^-1^ manure. Different lowercase letters on the top of vertical bars indicate significant differences between treatments (*P* <0.05).
